# Transformer with difference convolutional network for lightweight universal boundary detection

**DOI:** 10.1371/journal.pone.0302275

**Published:** 2024-04-16

**Authors:** Mingchun Li, Yang Liu, Dali Chen, Liangsheng Chen, Shixin Liu

**Affiliations:** 1 College of Information Engineering, Shenyang University, Shenyang, China; 2 College of Information Science and Engineering, Northeastern University, Shenyang, China; Khalifa University, UNITED ARAB EMIRATES

## Abstract

Although deep-learning methods can achieve human-level performance in boundary detection, their improvements mostly rely on larger models and specific datasets, leading to significant computational power consumption. As a fundamental low-level vision task, a single model with fewer parameters to achieve cross-dataset boundary detection merits further investigation. In this study, a lightweight universal boundary detection method was developed based on convolution and a transformer. The network is called a “transformer with difference convolutional network” (TDCN), which implies the introduction of a difference convolutional network rather than a pure transformer. The TDCN structure consists of three parts: convolution, transformer, and head function. First, a convolution network fused with edge operators is used to extract multiscale difference features. These pixel difference features are then fed to the hierarchical transformer as tokens. Considering the intrinsic characteristics of the boundary detection task, a new boundary-aware self-attention structure was designed in the transformer to provide inductive bias. By incorporating the proposed attention loss function, it introduces the direction of the boundary as strongly supervised information to improve the detection ability of the model. Finally, several head functions with multiscale feature inputs were trained using a bidirectional additive strategy. In the experiments, the proposed method achieved competitive performance on multiple public datasets with fewer model parameters. A single model was obtained to realize universal prediction even for different datasets without retraining, demonstrating the effectiveness of the method. The code is available at https://github.com/neulmc/TDCN.

## 1. Introduction

Boundary detection is a fundamental problem in computer vision [1]. Given an image, the purpose is to provide the corresponding binary result and reveal which pixels are boundaries. These boundaries are mostly related to the visually salient objects in an image [2]. Therefore, boundary detection plays an important role in various vision tasks, such as image segmentation [3], object detection [4], and image inpainting [5]. Considering its wide range of applications, it has long been a focus of computer vision research [6].

In a general sense, boundaries are often accompanied by dramatic changes in brightness, color, and texture [7]. Traditional methods mostly focus on local brightness and texture information, and boundary detection algorithms are designed through first-order or second-order gradients between pixels, such as the Sobel operator [8]. With the popularity of deep learning, many researchers have designed hierarchical convolutional networks to extract deep abstract features in addition to local features. For example, HED [9] designed a five-stage convolutional network to extract low- and high-scale features simultaneously to achieve the boundary detection of objects of different sizes. Subsequently, larger-scale deep models were proposed to achieve higher performance scores [10–15]. However, as a low-level vision task, boundary detection itself does not have clear application significance. To perform subsequent tasks better, the boundary detection method should be sufficiently simple and applicable to a variety of scenarios [6]. Lightweight unified yet efficient boundary detection remains an open problem.

Recently, considering the inherent shortcomings of convolutional networks in capturing long-distance dependencies, multiple patch-based methods have been proposed. Among them, transformers have attracted considerable attention for computer vision tasks because of their strong representation capabilities and efficiency [16, 17]. Their performance is comparable to that of popular convolutional neural networks (CNNs) and has prompted researchers to attempt to solve vision problems based on transformers [18–24]. In particular, in image segmentation tasks, transformers have been extensively applied to natural images [25], medical images [26], and remote-sensing images [27]. Moreover, in addition to the transformer-based methods, as an alternative to CNNs, multilayer perceptron-based (MLP) model has gradually become the research focus in solving vision tasks. For example, TGMLP U-Net [28] proposed an effective yet lightweight medical image segmentation model based on MLP, which encodes three-dimensional spatial features to enhance the sensitivity of position information. Inspired by these excellent works in image segmentation tasks, boundary detection is also a type of dense prediction, and the incorporation of these novel network structures into boundary detection models merits further research.

However, directly using the currently developed transformer or multilayer perceptron makes it difficult to satisfy the requirements of lightweight boundary detection tasks. Essential differences exist between boundary detection and image segmentation tasks. Semantic segmentation usually requires an encoder-decoder or a deeper backbone to extract more abstract and global features [29]. In contrast, boundary detection focuses more on local features and is a low-level visual task. Target boundaries are typically continuous and locally sensitive. Although some methods introduce local priors to improve the localization ability [28] or enhance regional consistency [30] in segmentation tasks, considering that the actual boundaries are only one pixel wide, these methods may not be completely suitable for the current tasks. Therefore, the use of these novel structures to implement boundary detection requires adjustments. To the best of the authors’ knowledge, transformer-based EDTER [31] and DiffusionEdge [32] have been proposed and have successively refreshed the score record. Although these transformers still rely on large backbones, their specific designs for enhancing the local features and generating crispness boundaries are impressive. This shows that an appropriately adjusted transformer has powerful representation learning capabilities for boundary detection tasks.

In this study, the focus was on using a lightweight transformer to solve the boundary detection problem. Specific adjustments were necessary to help the transformers obtain boundary-related knowledge under limited parameters. To solve this problem, gradient information was incorporated into a boundary-detection transformer. Inspired by PiDiNet [33], a learnable difference convolution [34] was introduced into the backbone to enhance the transformer. Compared with the attention between distant patches in the raw attention mechanism [35], the attention between patches and local potential trends appears to be more meaningful. Therefore, a novel boundary-aware self-attention mechanism was explored, and a loss function was designed to guide the focus of the proposed model. It is believed that such an adjustment is more suitable for lightweight models for boundary detection tasks.

**Fig 1:**
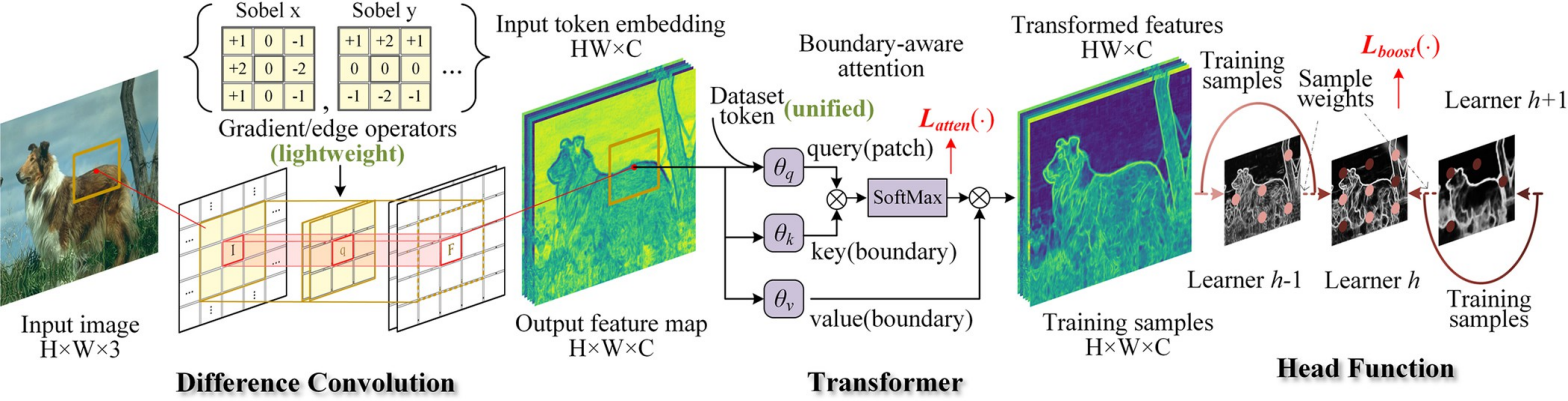
Key technologies of the proposed method.

A vision transformer structure was devised to solve the boundary detection problem, as shown in Fig 1. To adapt to the boundary detection task (low-scale vision task), a difference convolution network in the token acquisition stage, a boundary-aware attention mechanism in the transformer, and a boosting multiscale training strategy for the head function were designed. These designs yielded interesting results. For example, the convolution network with a gradient/edge operator significantly reduced the number of training parameters required. By viewing a class token as a dataset token, a model can simultaneously serve as multiple datasets at the same time. The contributions can be summarized as follows.

1. A new boundary-aware self-attention mechanism was developed. In this mode, the key-value pair is derived using directional filtering, and its similarity to the original token is measured. Based on this, an attention loss function was used that can explicitly guide the model to where more attention should be paid.
2. A convolution network combined with an edge operator was applied to extract absolute gradient information. By deeply fusing the convolution network and edge operators in a cardinality style, more boundary-related information can be extracted effectively.
3. A boosting strategy was designed for a multihead deep network based on ensemble learning. By dynamically updating the sample weights, multiple head functions with their respective scales can develop their strengths and avoid their weaknesses.

## 2. Related works

### 2.1 Boundary detection methods

From the well-known Canny operator [36] based on rules to CNNs based on learning, boundary detection tasks have existed for a long time. They are important for high-level vision tasks [6] and have a wide range of applications [37]. Early researchers tended to use local first-order or second-order gradients between pixels to design boundary detection algorithms, such as the Sobel operator and Canny algorithm. Considering that pixel-level gradients are not sufficiently robust, researchers have extracted local statistical features of brightness, color, and texture to enhance detection performance, similar to gPb [38], SCG [39], and OEF [40]. These rule-based methods are relatively simple; however, owing to the lack of context and semantic image cues, the detected results contain many meaningless edges in the background.

With the rise of deep learning, researchers have begun to use CNNs to solve boundary detection problems. For example, DeepEdge [41] employs a multilevel CNN to extract object-aware cues that are used to filter redundant points from the Canny operator. DeepContour [42] first partitions images into small patches and uses a hierarchical network to learn the boundary subclasses. Specifically, an end-to-end boundary detection method, HED [9], was proposed, which is remarkable. The method involves designing a hierarchical multiscale convolutional network structure to extract local and deep features simultaneously to achieve effective boundary detection for objects of different sizes. Subsequently, RCF [43] extracts more features from a multiscale backbone to achieve better detection results. To obtain diverse convolutional features, BDCN [13] uses a larger model with a bidirectional cascade structure to guide the training of each layer. Most recently, considering that images in benchmark datasets have multiple annotations, researchers have begun to pay more attention to the rationality of labels and have proposed alternative datasets [14] or refinement methods to produce crisp boundaries [2, 44]. In contrast, BetaNet [45] and UAED [15] employ model uncertainty strategies based on controversial annotations by mapping a beta and a Gaussian distribution, respectively.

The difference between the present study and most previous ones lies in the need for the model to be lightweight and universal. In pursuit of better performance or complicated functionality, researchers are currently dedicated to designing increasingly larger models for extracting richer features. However, as a low-level vision task, boundary detection itself does not have clear application significance. To perform subsequent tasks better, the boundary detection method should be sufficiently simple and applicable to a variety of scenarios. Although PiDiNet [33] attempts to incorporate local differential operators into convolutional networks to reduce the number of model parameters, there is still room for improvement because of the lack of sufficient global features. Instead, considering the powerful representation learning ability of recent transformers, a novel backbone structure was built in this study that fuses the transformer and CNN to improve performance further under limited parameters.

### 2.2 Vision transformer and variants

The transformer was first proposed for use in natural-language processing and then expanded to vision tasks. The pioneering work was the vision transformer [17]. Compared with convolution, it is better at capturing long-distance characteristics. Subsequently, researchers have begun to improve the transformer structure to adapt it to vision tasks. Its variants mainly introduce prior knowledge, such as hierarchy [46] and locality [47, 48]. For example, Swin [25] employed a patch-merging module for hierarchy and window mechanisms to enhance locality. Moreover, some researchers have attempted to modify the self-attention mechanisms, as in PVT [30] and ViL [49]. Others were concerned with such details as position embedding [50], training strategy [51], and fusing convolution [52]. Recently, the transformer-based EDTER [31] was proposed for boundary detection tasks. It constructs a two-stage model to extract global context features and local features for refinement. Subsequently, a diffusion probabilistic model [32] used for boundary detection was designed to generate crisp boundaries, and it achieved the highest performance score to date under the transformer backbone. Although transformers mostly rely on large backbones and massive data, the performances of these methods demonstrate their effectiveness in boundary detection tasks.

The present work was inspired by these pioneering studies but is significantly different in three respects. The adjustment of the transformer aims to achieve lightweight and unified boundary detection. First, from the perspective of the backbone design, multiple differential convolution operators that provide gradient information are introduced to help the transformer obtain boundary-related clues using fewer parameters. Second, a novel boundary-aware attention and loss function was developed to highlight the boundary information rather than a patch-based mechanism. Finally, considering the universality of the boundary detection task, dataset embedding was introduced in the transformer so that a single model can be applied to multiple datasets simultaneously. With these adjustments, the model can achieve lightweight unified yet efficient boundary detection.

## 3. Methodology

**Fig 2:**
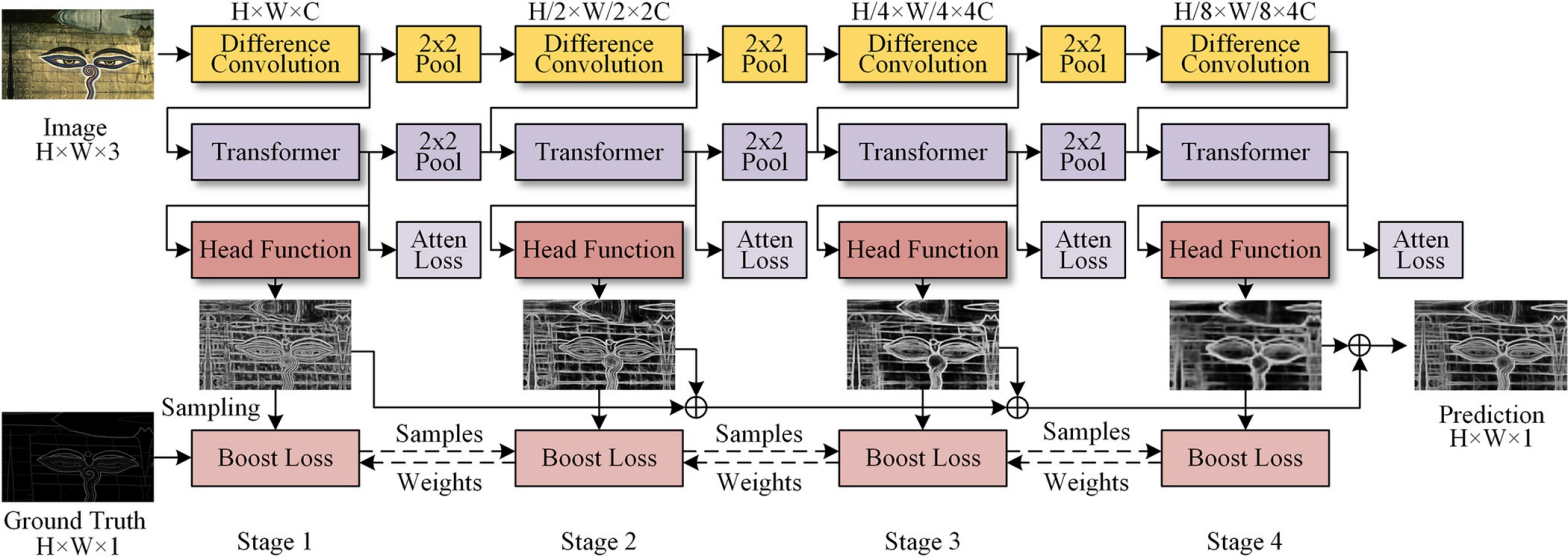
TDCN structure.

In this section, the proposed transformer with difference convolution network (TDCN) is described in detail. It consists of four stages, as illustrated in Fig 2. At each stage, the convolution, transformer, and head functions are performed sequentially, and each of these is introduced. For simplicity, the important notations and their definitions are listed in Table 1.

**Table 1:** Important notations and the corresponding definitions in this work.

Notation	Definition	Notation	Definition
(*x*,*y*)	image-label pair	(*x*_*i*_,*y*_*i*_)	*i* th index in image-label pair
H, W	height and width of image	*F*	feature map
*s*	stage (scale) index of model	*θ*	learnable parameters of model
C_*s*_	channel number in stage s	C_head_	channel number in head function
*p*_*i*_	*i*th patch embedding	*p*_*ds*_	dataset embedding
${\tilde{p}}_{i}$	transformed *i*th patch embedding	${\tilde{p}}_{ds}$	transformed dataset embedding
*b*_*j*_	*j*th boundary embedding	*yb*_*j*_	*j*th index in boundary ground truth
*T*	total number of base learners	*α*	balance coefficient

### 3.1 Difference convolution module

Difference convolution is essentially the fusion of edge operators with convolution. Considering the relationship between gradient and boundary [53], it is worthwhile to consider explicitly introducing gradient information. In a pioneering study on face antispoofing [34], raw convolution added a central difference when obtaining facial information. In PiDiNet [33], the angular and radial differences are also considered. In the present study, more effective edge operators were introduced to capture richer gradient information.

**Fig 3:**
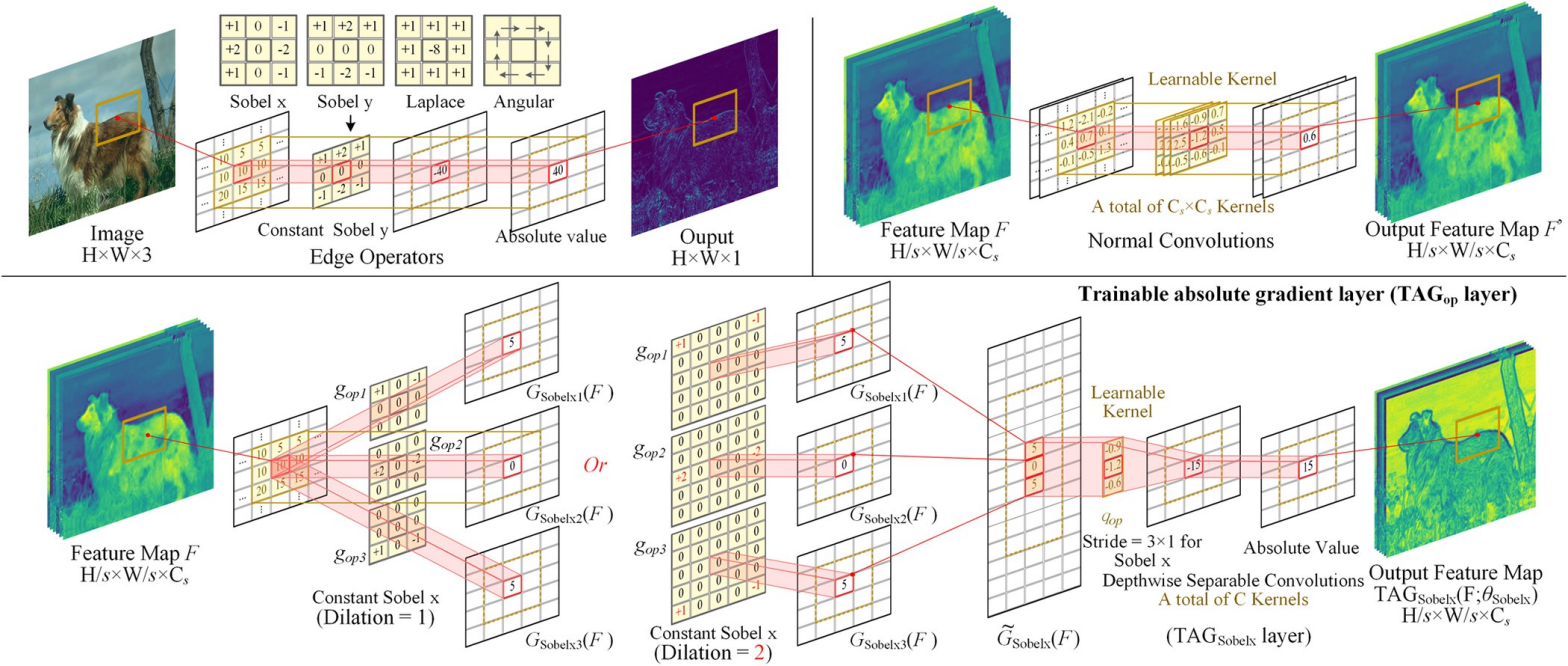
Edge operators, normal convolutions, and trainable absolute gradient (TAG) layer.

First, the Sobel [8] and Laplace operators, which capture the first- and second-order gradients, are considered. An angular operator [54] related to the textural features is also considered, as shown in Fig 3 (upper left). For red-green-blue (RGB) images with a size of H×W×3, because the boundary detection task only considers the intensity of the gradient, the absolute value is extracted directly. These are essentially 3 × 3 spatial filters, but the specific kernel function is different. However, the edge operator operates only on raw images. When the operating object becomes a feature map in deep learning, to improve the performance further, one can increase the learning ability of these edge operators by introducing trainable parameters. Inspired by previous research [33], these kernel functions are integrated into convolution networks. In other words, the spatial difference is added before the normal convolution kernel, as shown in Fig 3 (lower). Supposing *F* in stage *s* is a given feature map of size H/*s*×*W*/*s*×C_*s*_, it can be implemented in the following two steps.

$$G_{op}(F)=[G_{op1}(F),G_{op2}(F),\cdots ,G_{opn}(F)],$$ (1)

$${TAG}_{op}(F;\theta_{op})=|\theta_{op}∙G_{op}(F)|=|\theta_{op}*{\tilde{G}}_{op}(F)|,$$ (2)

where *TAG*_*op*_(·) is the trainable absolute gradient (TAG) layer for edge operator *op* with parameters *θ*_*op*_,*G*_*opi*_(*F*) = *g*_*opi*_ * *F* is a subedge operator with kernel *g*_*opi*_ and a total of *n*,* is the convolution symbol, · is the dot product between vectors, and ${\tilde{G}}_{op}(F)$ means the stitched map based on vector *G*_*op*_(*F*).

In Fig 3, the raw edge operator *G*_*op*_(·) is split to subkernel *g*_*opi*_.More templates are proposed than before. Because multichannel maps are operated upon, richer features can be extracted. Moreover, the number of suboperators for different edge operators is specific. For example, for Sobelx or Sobely, it is split into three suboperators in the transverse or longitudinal direction. For Laplace, it would have eight suboperators.

**Fig 4:**
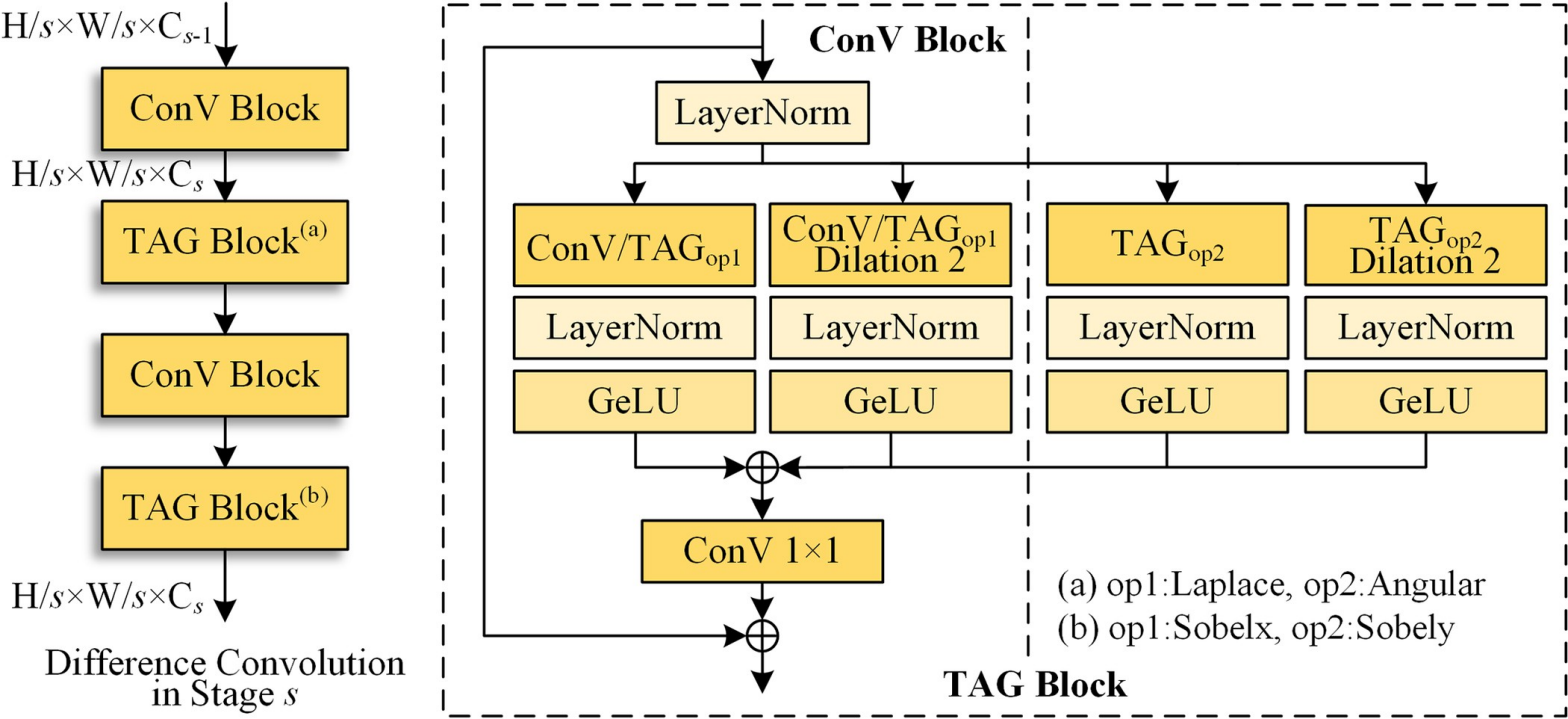
Structure of difference convolution module.

In essence, if one ignores the specific edge operation (Sobel) in this study, it is similar to pixel difference convolution [33], but with an absolute value operation. Moreover, some “tricks,” such as depthwise separable convolutions [55] and dilated convolutions [56], are used. For the dilated convolution version TAG layer, one can expand the scale of edge operator *G*_*op*_(·) from 3 × 3 to 5 × 5 to enhance the receptive field, as shown in Fig 3 (lower). The structure of the overall difference convolution module based on the TAG layer is shown in Fig 4.

Specifically, the proposed difference convolution module consists of four blocks: two are related to normal convolution, and the other two are related to the TAG layer. The first and third blocks have the same structure, including layer normalization [57], two depthwise separable convolutions, Gaussian error linear unit (GeLU) activation, and convolutions with a 1 × 1 kernel. Similarly, when it comes to TAG blocks (the second and fourth), the two convolutions are replaced with depthwise separable TAG layers. The first TAG layer is based on the Laplace and angular operators, whereas the last TAG layer is based on the Sobel operator.

In summary, the proposed difference convolution is the interleaved structure of the normal convolution and the TAG block. It helps the network capture locality and gradient information simultaneously. The final output of the feeding transformer is from the TAG block. It explicitly contains richer gradient information. Compared with vanilla split patches, such input tokens for transformers are important in the boundary detection task, which is helpful in obtaining and transforming correct and valuable features that require attention.

### 3.2 Transformer block and boundary-aware attention

In this section, we introduce boundary-aware attention into the TDCN. First, typical self-attention is reviewed. If a set of signals p = [*p*_1_,*p*_2_⋯*p*_*n*_,*p*_*ds*_] with length *n*+1 (the last one is the dataset token *p*_*ds*_, while in previous research [17] it was used as a class token for classification) is input, and the self-attention mechanism produces output $\tilde{p}=[{\tilde{p}}_{1},{\tilde{p}}_{2}\cdots {\tilde{p}}_{n},{\tilde{p}}_{ds}]$ of the same size based on the inner similarity criterion between *p*_*i*_, the output signal ${\tilde{p}}_{i}$ can be calculated as

$${\tilde{p}}_{i}=\frac{1}{N(p_{i})}\sum_{\forall j}l_{PPS}(i,j)M(q(p_{i};\theta_{q}),k(p_{j};\theta_{k}))v(p_{j};\theta_{v}),$$ (3)

where *M*(∙,∙) is the pairwise similar measurement function between vectors, *q*(∙),*k*(∙),*v*(∙) are mappings, $N(p_{i})=\sum_{\forall j}l_{PPS}(i,j)M(q(p_{i};\theta_{q}),k(p_{j};\theta_{k}))$ is used as normalization, and $l_{PPS}(∙,∙)$ is an indicator that only the pair belonging to set PPS returns 1.

**Fig 5:**
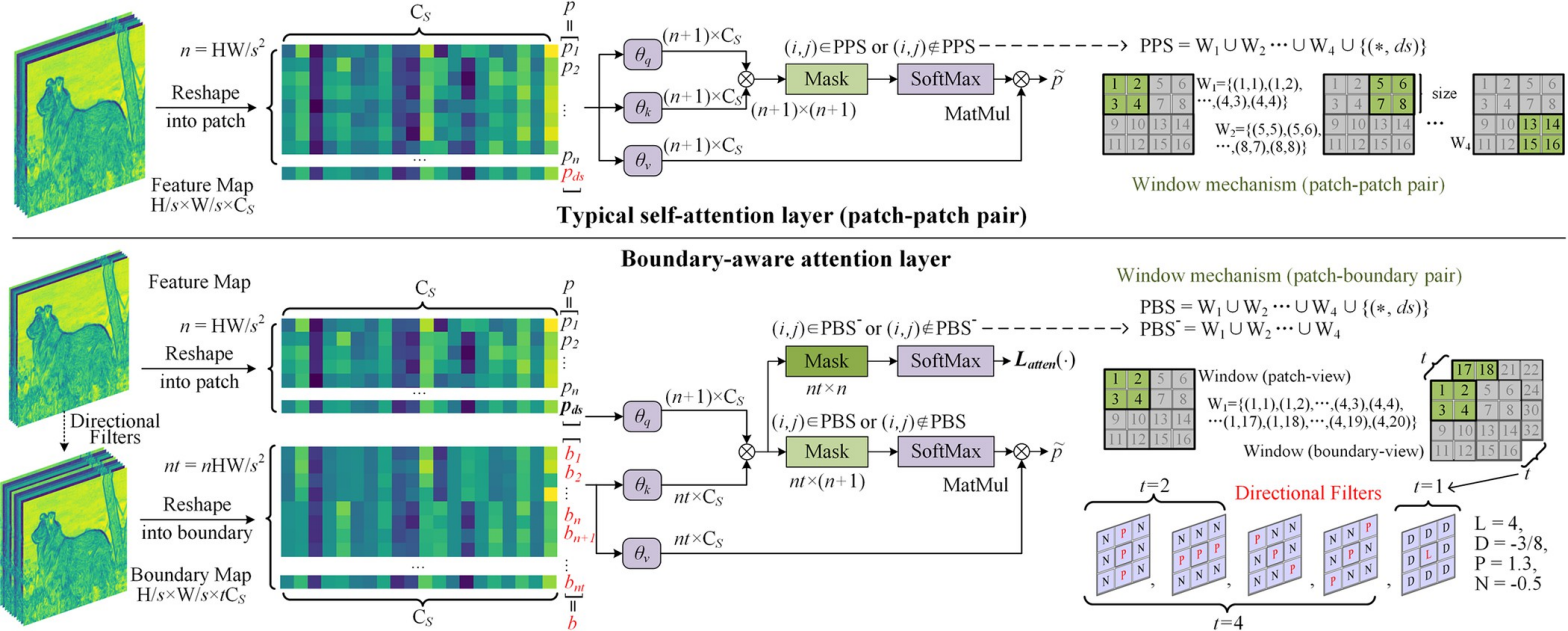
Typical self-attention layer and proposed boundary-aware attention layer.

In Eq (3), input signal *p* is a token embedding. In vision problems, a more specific form is patch embedding [17]. The pairwise function can be formulated as a dot product, $M(q(p_{i};\theta_{q}),k(p_{j};\theta_{k}))=\exp\left(q(p_{i};\theta_{q})k(p_{j};\theta_{k})\right)$. The *q*(∙),*k*(∙), and *v*(∙) with parameters *θ*_*q*_,*θ*_*k*_,*θ*_*v*_ are linear projection, and the outputs are called “query,” “key,” and “value.” Moreover, indicator function $l_{PPS}(∙,∙)$ is introduced in Eq (3) to cover general situations. For the raw vision transformer, the patch-patch set (PPS) covers all possible patch pairs. However, in some studies aimed at boosting locality, the set might only include adjacent patch pairs within the window, such as Swin [25]. A typical self-attention layer with a window configuration is shown in Fig 5 (upper).

Careful examination of Fig 5 (top) reveals that the existing self-attention mechanism is based on the similarity of different patches *p*. Using a patch as the basic unit is effective for many vision tasks, such as classification. Moreover, for segmentation and detection tasks, PVT [30, 58] introduces spatial-reduction attention to enhance regional consistency using patch-square pairs. When the problem comes to boundary detection, as in this study, it can be further improved by introducing inductive bias. In particular, the boundary should be continuous and directional. Compared with the use of a patch as the basic unit, the boundary seems to be a better alternative. The proposed scheme is as follows.

$${\tilde{p}}_{i}=\frac{1}{NB(p_{i})}\sum_{\forall j}l_{PBS}(i,j)M(q(p_{i};\theta_{q}),k(b_{j};\theta_{k}))v(b_{j};\theta_{v}),$$ (4)

Where *b*_*j*_ is the extracted boundary feature vector, $l_{PBS}$ indicates the indicator function dominated by the patch-boundary set (PBS), and $NB(p_{i})=\sum_{\forall j}l_{PBS}(i,j)M(q(p_{i};\theta_{q}),k(b_{j};\theta_{k}))$.

This new self-attention mechanism is called “boundary-aware attention,” as shown in Fig 5 (lower). Compared with raw self-attention, the similarity between patch-boundary pairs instead of patch-patch pairs is measured. The set PBS contains patch-boundary pairs within the specified window size (such as 2 in Fig 5) and patch-dataset token pairs {(*,*ds*)}. In other words, the key *k*(*b*_*j*_) and value *v*(*b*_*j*_) in Eq (4) are derived from the boundary embedding *b* = [*b*_1_,*b*_2_⋯*b*_*n*_,*b*_*n*+1_,*b*_*n*+2_⋯*b*_*nt*_], while the query *q*(*p*_*i*_) remains unchanged, which is still based on the original patch embedding *p*. Boundary embedding can be obtained by several (*t*) spatial directional filters. The total number *nt* f boundary embeddings is an integral multiple of the patch embedding *n*, depending on the number of filters (in Fig 5, lower). In this manner, one can capture the relationship between the patch and boundary to achieve boundary-sensitive attention.

Attention loss is introduced. Benefiting from boundary-aware attention, one can explicitly guide the focus of the model. In raw self-attention, it is difficult to determine which patches should be focused on. However, once the objects become boundaries using filters, a loss can be set to force the patch to focus on meaningful boundaries. A set containing only patch-boundary pairs is denoted as PBS^−^. For a patch-boundary pair (*i*,*j*)∈PBS^−^, the loss is

$$L_{a}(p_{i},b_{j},y_{i},{yb}_{j})=-\frac{M(y_{i},{yb}_{j})}{NB(y_{i})}\log\left(\frac{M(q(p_{i};\theta_{q}),k(b_{j};\theta_{k}))}{NB(p_{i})}\right),$$ (5)

Where *y*_*i*_,*y*_*bj*_ are the corresponding patch *i* and boundary *j* directly extracted in ground truth, and the normalization $NB(y_{i})=\sum_{\forall j}l_{{PBS}^{-}}(i,j)M(y_{i},{yb}_{j})$.

In Eq (5), *y*_*bj*_ can be indexed and obtained by directional filtering of ground truth *y*. If (*y*_*i*_,*y*_*bj*_) is viewed as a label, the loss is similar to cross entropy. This is related to the patch direction, according to the boundaries involved. Here, the label is a real value vector rather than one-hot. In essence, the attention loss implies supervised boundary information based on boundary-aware attention. This encourages paying more attention to the boundary consistent with its own direction and ignoring others.

There are multiple attention modules in the TDCN. Each stage has an attention module, for a total of four. Unlike the original scale (first stage) with supervision, (*y*_*i*_,*y*_*bj*_) can be obtained directly. To obtain the supervision pair for other scales, downsampling must also be performed several times on the ground truth. The attention loss for stage *s* is

$$L_{Atten}^{s}(x,y;\theta^{s})=\sum_{i=1}^{WH{/s}^{2}}w({{Po}^{s}(y)}_{i})\sum_{\forall (i,*)\epsilon {PBS}^{s-}}L_{a}({{DC}^{s}(x;\theta^{s})}_{i},{{DCB}^{s}(x;\theta^{s})}_{*},{{Po}^{s}(y)}_{i},{{PoB}^{s}(y)}_{*}),$$ (6)

Where *x* is input image with size of *HW*,*y* is the ground truth with the same size, *Po*^*s*^(∙),*PoB*^*s*^(∙) are the pooling operation of *s* times to obtain a patch-boundary pair for *y*, the definition of *DC*^*s*^(∙),*DCB*^*s*^(∙) is the same, except that there are parameters *θ*^*s*^ to be optimized, set PBS^*s*−^ is the patch-boundary set for stage *s*, and *w*(∙) is employed to produce weight.

Combined with Eq (5), *DC*^*s*^(∙) can be seen as a feature extractor for input *x* and *p*_*i*_ = *DC*^*s*^(*x*)_i_ Moreover, the weight of one patch *Po*^*s*^(*y*)_*i*_ is determined by the stage and category. It is desirable to balance the attention loss across different stages and categories. This can be expanded as follows.

$$w({{Po}^{s}(y)}_{i})=\left\{ \begin{array}{c} s^{2}∙\frac{{NumN}^{s}}{{Total}^{s}},\;if\;{{Po}^{s}(y)}_{i}=+1\\ s^{2}∙\frac{{NumP}^{s}}{{Total}^{s}},\;if\;{{Po}^{s}(y)}_{i}=-1 \end{array}\right.,$$ (7)

Where Total^*s*^ is the total number of patches in stage *s*, and NumP^*s*^ and NumN^*s*^ are the numbers of patches involved or not involved in the boundary, respectively.

**Fig 6:**
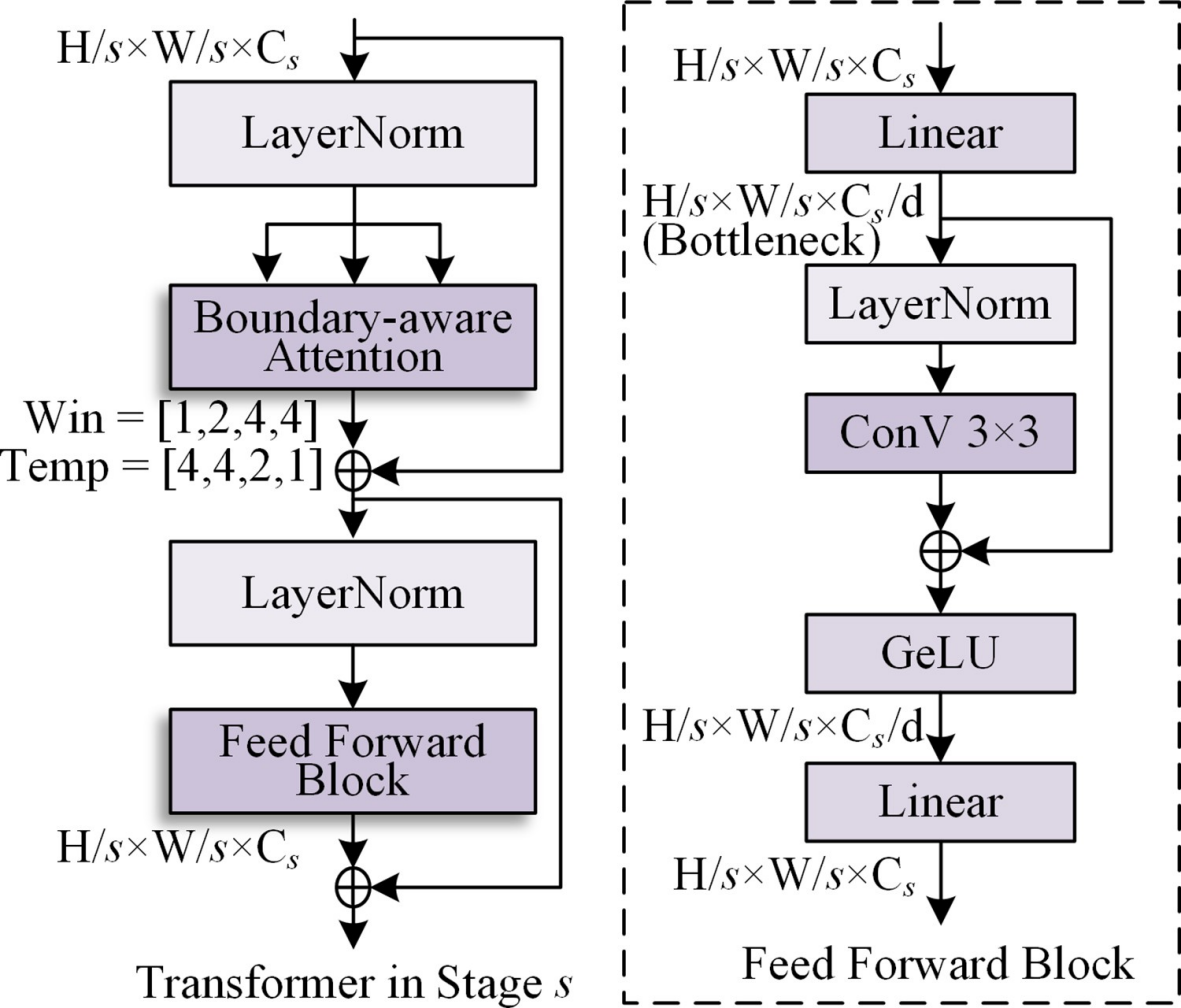
Transformer with boundary-aware attention.

Regarding the overall structure, unlike the previously discussed convolution, the configurations of the attention block in different stages are specific. The configuration mainly refers to the window size and number of templates *t*. The former determines the elements involved in set PBS^*s*^. The latter is the number of spatial directional filters, which is related to the loss calculation. The overall structure of the transformer and the specific configuration of the attention in different stages are shown in Fig 6.

The overall structure is the same as that of a typical transformer [17]. Regarding the configuration of the attention block, with the deepening of the network, the window size is larger, and the number of templates is smaller. The windows of the four stages *s* are set to 1, 2, 4, and 4, respectively. When the window is set to 1, patch *p*_*i*_ only considers the similarity measurement with the boundaries *b*_*i*1_,*b*_*i*2_⋯*b*_*it*_ centered on the same position. The templates *t* of the four stages *s* are set to 4, 4, 2, and 1. All the filters in Fig 5 are used when the template is 4. When it is 2, only the horizontal and vertical directional filters are considered. When it is 1, a difference filter is used without direction preference. The details are shown in Fig 5.

Smaller patches are necessary for dense pixel-level predictions. In this network, a patch in the first stage is a pixel. To boost the locality and positioning ability, in the initial stage (the first two stages), all four types of filter and a smaller window size are used. With an increase in scale, the boundaries are often more blurred and difficult to locate accurately after several downsampling cycles. Therefore, fewer filters are used to save computational power, and a larger window size is employed to capture long-distance characteristics.

Finally, the transformer can be trained and make predictions on multiple datasets simultaneously. Setting several dataset tokens *p*_*ds*_ and positional tokens according to the number of datasets is sufficient to realize a unified prediction. In other words, in the training phase, when the image comes from dataset A, one uses the first dataset token in the attention mechanism. If the image comes from dataset B, one chooses the second dataset token. The only task is to mix the multiple datasets. If these datasets have similar capacities, the random sampling of a mixed dataset could be simple and effective. During the test, tokens of the corresponding datasets were adopted. The parameter increase caused by these tokens was extremely small and could be ignored. Thus, a unified prediction was realized based on only one network.

### 3.3 Head function

**Fig 7:**
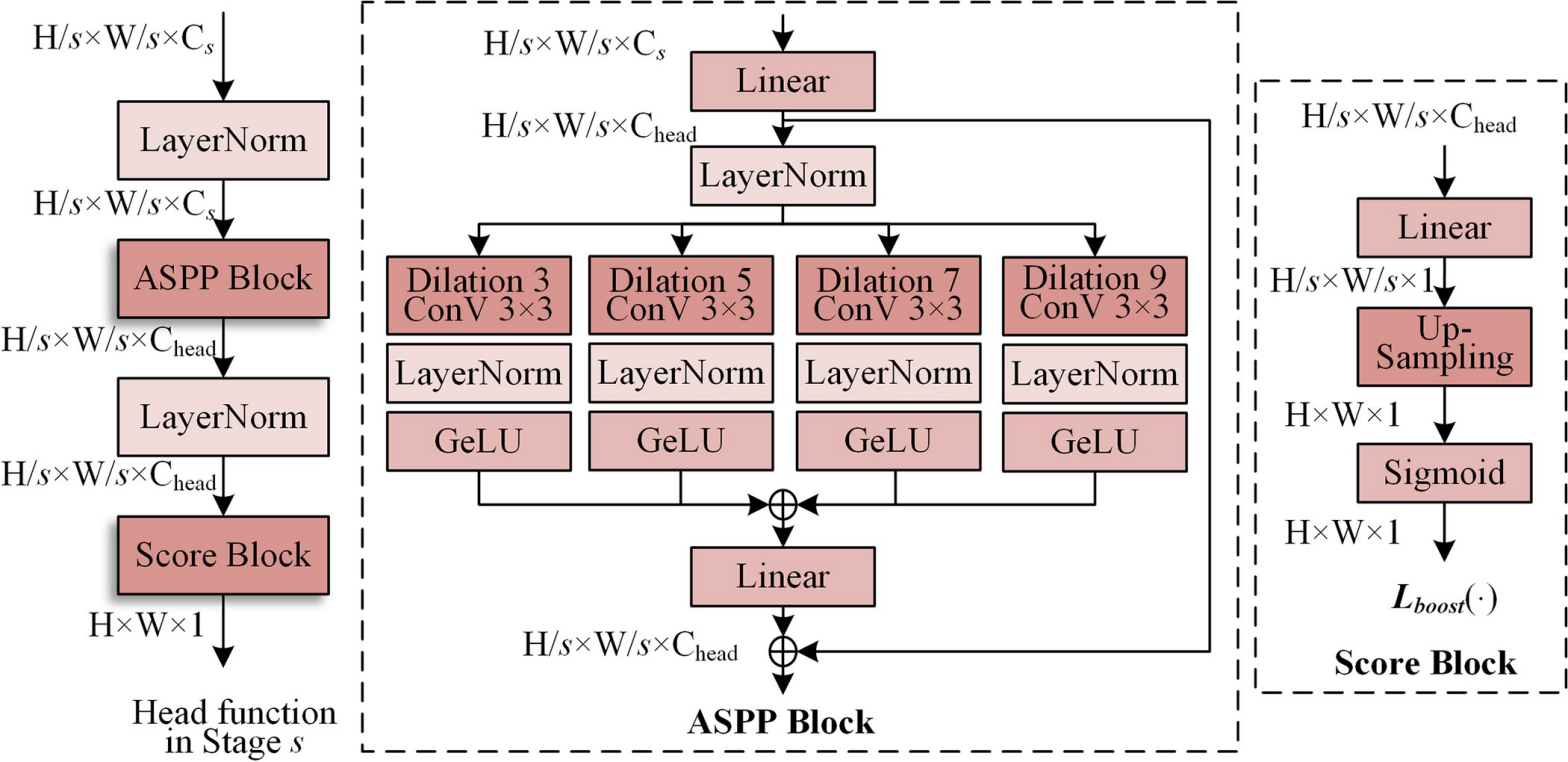
Structure of the head function.

In this section, the head function and boosting training strategy of the TDCN are introduced. The head function produces classification results based on the features extracted from the backbone (difference convolution and transformer). Specifically, the output is a probability map of the same size as the image. For the head function in different stages, additional upsampling may be required according to a specific scale. Its structure, inspired by atrous spatial pyramid pooling (ASPP) [29], is shown in Fig 7.

As Fig 7 shows, the head function consists of an ASPP block and a score block. In ASPP, a linear layer is designed for feature dimensionality reduction from C_*s*_ to C_head_. The following four dilation convolutions with multiple rates are performed in parallel and output to the normalization and activator layers. They are then fed into the linear layer for fusion. The score block is used to restore the raw scale and make predictions using upsampling and a sigmoid activator.

In the TDCN, there are four head functions. They have the same structure, except for the configuration of the upsampling layer. It is feasible to train each head function independently based on a unified ground truth. However, it is believed that the head function based on different-scale features can be responsible for the respective boundaries. The head function based on small-scale features should strengthen the positioning ability, and the head function based on large-scale features should focus on judging the authenticity of the boundary. In this study, the AdaBoost strategy [59] was used to train the base learners. It can be described as

$$H^{S}(x_{i})=\sum_{s=1}^{S}\beta^{s}h^{s}(x_{i};\theta^{s}),$$ (8)

$$(\beta^{s},\theta^{s})=\underset{\beta ,\theta }{\mathrm{argmin}}\sum_{i=1}^{N}exp(-y_{i}∙(H^{s-1}(x_{i})+\beta h^{s}(x_{i};\theta ))),$$ (9)

Where *H*_*s*_(∙) is the additive model from scale 1 to scale *S*,*H*^0^(∙) = 0 indicate the initial state, *h*^*s*^(∙) with lower-case letter s is the head function with parameter *θ*^*s*^,*β*^*s*^ is a coefficient, and (*x*_*i*_,*y*_*i*_) is a sampled data pair from a dataset of size *N*.

Eq (9) shows the logic of AdaBoost without normalization, which is based on an additive model under an exponential loss. When solving the parameter *θ*^*s*^ in *h*^*s*^(∙), previous base learners are fixed based on the forward stagewise algorithm. One can extract exp[‒*y*_*i*_ *H*^*s*‒1^(*x*_*i*_)] as the sample weight for data pair (*x*_*i*_,*y*_*i*_) to train the next learner. In this manner, the new learner can focus more on the samples that were misclassified by the previous ones.

In contrast to classic boosting based on homogeneous base learners, the head functions are performed using different scale features. They should be trained in a specific order, e.g., from a low to a high scale. First, the parameters involved in the first stage are trained. Because of the lack of semantic information, some regions may be incorrectly classified as having a low-scale head function. These samples are assigned more attention to train the following learners (high scale) with deeper and abstract features. Ideally, the samples that are difficult for low-scale learners are those that high-scale learners are good at, and vice versa. In this study, a bidirectional boosting strategy was used to train the network, as described below.

$$H^{I,E}(x_{i},y_{i})=wh(y_{i})+\sum_{s=I}^{E}\rho ∙\frac{1}{2}log(\frac{h^{s}(x_{i};\theta^{s})}{1-h^{s}(x_{i};\theta^{s})}),$$ (10)

$$\theta^{s}=\underset{\theta }{\mathrm{argmin}}\sum_{i=1}^{N}exp[-y_{i}∙\frac{1}{2}\log\left(\frac{h^{s}(x_{i};\theta )}{1-h^{s}(x_{i};\theta )}\right)-y_{i}∙(H^{1,s-1}(x_{i},y_{i})+H^{s+1,T}(x_{i},y_{i}))],$$ (11)

where *H*^*I*,*E*^(∙,∙) is an additive model from scale *I* to ending $E,H^{1,0}(∙,y_{i})=H^{T+1,T}(∙,y_{i})=wh(y_{i})$ means the initial stage, *wh*(∙) is the return category weight, *ρ* = 0.4 is a preset value, and *T* indicates the total number of base learners (in this study, *T* = 4).

Eqs (10) and (11) are displayed according to the logic of a probabilistic learner rather than a binary learner. The coefficients *β*^*s*^ no longer appear, and the predictions are log-transformed. Furthermore, *ρ* is introduced as step size or shrinkage. From Eq (11) one can see that, when the head function *h*^*s*^ is trained with parameters *θ*^*s*^, the sample weights are calculated by both the forward order *H*^1,*s*‒1^ and the reverse order *H*^*s*+1,*T*^ This can objectively guide the focus of the current scale head function *h*_*s*_ rather than a certain order. Moreover, in Eq (10), category weight function *wh*(∙) is introduced. It is primarily used to solve class imbalance problems. In other words, it can be understood as a more reasonable sample weight initialization than the uniform initialization of the original boosting. It can be expanded further as

$$wh(y_{i})=\left\{ \begin{array}{c} log(\frac{Total}{NumN}),\;if\;y_{i}=+1\\ log(\frac{NumP}{\pi ∙Total}),\;if\;y_{i}=-1 \end{array},\right.$$ (12)

Where *π* = 1.1 is a preset value to control the preferences, Total is the total number of samples, and NumP *and* NumN are the numbers of positive and negative samples, respectively.

Finally, a specific loss function is provided for Eq (11) in deep-network logic. Unlike the decision tree in the raw AdaBoost, a deep network requires iterative optimization based on the backpropagation algorithm. When the raw AdaBoost trains the next base learner, the previous learners are fixed. However, this case does not have such conditions. Therefore, the optimization of the deep network cannot be guaranteed, and the boosting strategy is directly implemented repeatedly in an iterative manner based on the loss function

$$w_{i}=\gamma \frac{exp[-y_{i}H^{1,s-1}(x_{i},y_{i})]}{{NH}^{1,s-1}(x,y)}+(1-\gamma )\frac{exp[-y_{i}H^{s+1,T}(x_{i},y_{i})]}{{NH}^{s+1,T}(x,y)},$$ (13)

$$L_{Boost}^{s}(x_{i},y_{i},w_{i};\theta^{s})=-w_{i}(l(y_{i}=1)log(h^{s}(x_{i};\theta^{s}))+l(y_{i}=-1)log(1-h^{s}(x_{i};\theta^{s}))),$$ (14)

Where *γ*~Bernoulli(0.5) is a random binary variable, the normalization ${NH}^{1,s-1}(x,y)=\sum_{i=1}^{N}exp[-y_{i}H^{1,s-1}(x_{i},y_{i})]/\sum_{i=1}^{N}exp[-y_{i}wh(y_{i})]$, and the definition of *NH*^*s*+1,*T*^(*x*,*y*) is the same.

Here, the binary variable *γ* following a Bernoulli distribution is introduced. For a data pair (*x*_*i*_,*y*_*i*_), only one sequence (*H*^1,*s*‒1.^ or *H*^*s*+1,*T*^) is activated. It is believed that the differentiated samples help improve the diversity of the method. Moreover, Eq (14) is equivalent to the binary cross-entropy loss function with sampling weight *w*_*i*_. It is an approximation for the exponential loss in Eq (11), considering that the population minimizers of both are located in true probabilities. This is also consistent with the algorithm flow of real AdaBoost [59]. In summary, combined with the proposed attention loss, the overall loss function for the TDCN is provided as shown below.

$$L(x,y;\theta )=\sum_{s=1}^{4}\alpha L_{Atten}^{s}(x,y;\theta^{s})+\sum_{s=1}^{4}\sum_{i=1}^{WH}L_{Boost}^{s}(x_{i},y_{i},w_{i};\theta^{s}),$$ (15)

Where *α* = 20 is used to balance the two loss functions.

The network is trained based on the fused loss function in Eq (15). The $L_{Boost}^{s}(∙)$ is involved in the head functions, while $L_{Atten}^{s}(∙)$ appears in the transformer. Moreover, although we use the same notation *θ*_*s*_, the number of parameters in $L_{Boost}^{s}(∙)$ is always larger than that of $L_{Atten}^{s}(∙)$ because the latter does not involve the parameters in the head function. During testing, the results of the four head functions are directly averaged to obtain the final probability—for example, $TDCN(x_{i})=0.25\sum_{s=1}^{4}h^{s}(x_{i};\theta^{s})$.

## 4. Experiments

### 4.1 Datasets and criterion

Here, two datasets are mainly considered: BSDS [38] and NYUD [60]. These two datasets are widely used for evaluating boundary detection methods. BSDS mainly contains natural landscape images, whereas NYUD involves indoor images. BSDS has 300 images for training and 200 test images. For these training images, data augmentation, such as flipping (×2), rotation (×16), and scaling (×3), is considered. The Pascal datasets [61] containing natural images are also used as additional training images after the flipping augmentation (×2). A total of 49,006 (BSDS 300 × 96, Pascal 10,103 × 2) training images are used for natural image boundary detection. In addition, NYUD contains two types of image: RGB and depth images. The latter are obtained using a specific camera and contain indoor depth information. The training and test sets contain 795 and 654 images, respectively. Similarly, some image augmentation methods are considered, such as flipping (×2), rotation (×4), and scaling (×3). For NYUD, there are 19,080 (NYUD 795 × 24) training and 654 test images.

The criterion for evaluating the boundary detection method is the F1 score based on the P-R curve, which is also called the optimal image scale (OIS) and optimal dataset scale (ODS). Owing to the specific image sizes of the different datasets, the maximum tolerated distances between the predictions and ground truth are 0.0075 for BSDS and 0.011 for NYUD. According to convention, nonmaximal suppression and edge thinning are performed before evaluation. The specific configuration is consistent with that of a previous method [43] for a fair comparison.

### 4.2 Implementation details

The TDCN is a hierarchical network consisting of four stages. The channel numbers of the difference convolution and transformer in the four stages are C_1_ = 42, C_2_ = 84, C_3_ = 168, *and* C_4_ = 168 respectively, and the channel number of the head is C_head_ = 16. The configuration of the transformer primarily involves attention layers. The window sizes of attention in different stages are [1,2,4,4], and the numbers of templates are [4,4,2,1]. Moreover, the attention mechanism is a multihead style that boosts diversity. For trainable position embedding, relative position token embedding was adopted in stages 2–4. This means that the token is related to the relative position rather than the absolute position, which helps improve the smoothness of prediction.

For training, the Adam [62] optimizer is used with a learning rate of 0.002. The batch size is set to 10 images, and the preset coefficient *π* in Eq (12) is selected as 1.1. One thing to note about dataset BSDS is that its ground truth is derived from more than one human. This causes the ground truth to be an average with real value. To calculate the boost loss, it is converted into a binary graph. Here, those parts greater than 0.3 are defined as the true boundaries, those between 0.3 and 0 as the fuzzy boundaries, and the rest equal to 0 as the background. Moreover, random initialization is employed for this network. When a single dataset (such as BSDS or NYUD) is trained, the final network is obtained after seven epochs, and the learning rate in the last epoch is divided by 10. When multiple datasets are trained simultaneously, the total number of epochs is set to 11. The learning rate is divided by 10 twice in the ninth and the last epoch, respectively.

### 4.3 Performance on BSDS and NYUD

**Fig 8:**
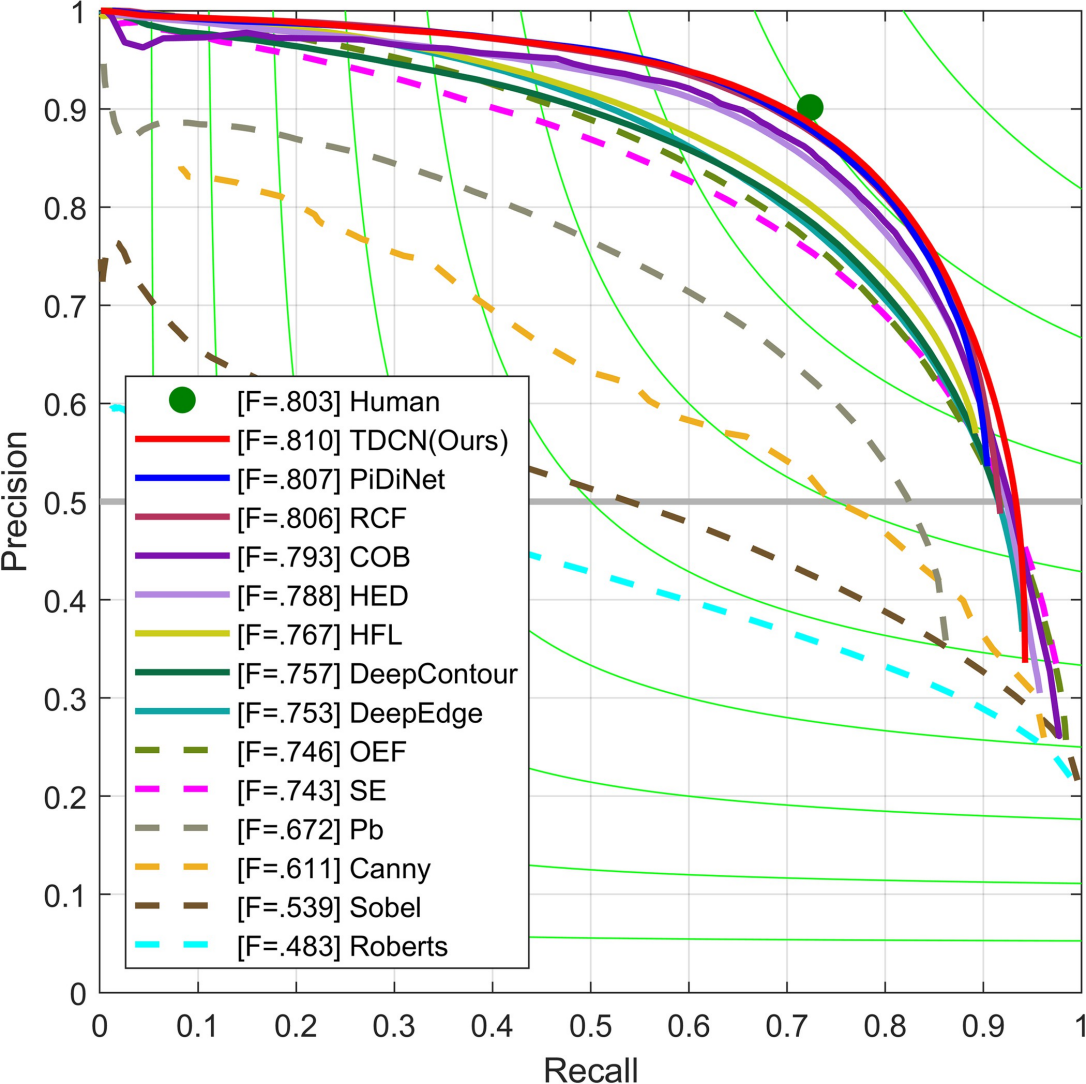
Precision-recall curves on BSDS dataset.

First, the method was evaluated using BSDS. Other methods were also considered for comparison. The other methods were divided into rule-based and deep-learning-based methods. The latter mainly refers to CNNs. Because each image in the BSDS is annotated by more than one person, human performance is also listed based on the previously discussed criterion. The quantization results and the corresponding precision-recall curves were provided directly based on the predictions, as shown in Table 2 and Fig 8.

**Table 2:** Performances on BSDS.

Method	ODS	OIS	Basis
Human	.803	.803	-
Canny [36]	.611	.676	Rule-based
Pb [7]	.672	.695	Rule-based
SCG [39]	.739	.758	Rule-based
SE [63]	.743	.763	Rule-based
OEF [40]	.746	.770	Rule-based
DeepEdge [41]	.753	.772	CNN
DeepContour [42]	.757	.776	CNN
HFL[10]	.767	.788	CNN
PiDiNet-up [2]	.780	.793	CNN
CHRNet [44]	.787	.788	CNN
CEDN [64]	.788	.804	CNN
FINED-Inf [65]	.788	.804	CNN
HED [9]	.788	.808	CNN
DeepBoundary [66]	.789	.811	CNN
FINED-Train [65]	.790	.808	CNN
COB [11]	.793	.820	CNN
CED [67]	.794	.811	CNN
AMH-Net [68]	.798	.829	CNN
BetaNet [45]	.803	.822	CNN
RCF [43]	.806	.823	CNN
PiDiNet [33]	.807	.823	CNN
LPCB [12]	.808	.824	CNN
BDCN [13]	.820	.838	CNN
UAED [15]	.838	.855	CNN
EDTER [31]	.832	.847	Transformer
DiffusionEdge [32]	.834	.848	Transformer
TDCN (Proposed)	.810	.826	Transformer-CNN

Table 2 and Fig 8 show that TDCN achieved a score of 0.810 in ODS and a score of 0.826 in OIS. These performances are higher than those of many deep-learning methods, such as DeepEdge (ODS-753), HED (ODS-788), and RCF (ODS-806). This demonstrates the effectiveness of the proposed method. However, the TDCN scores are lower than those of some other methods, such as EDTER (ODS-0.832) and UAED (ODS-0.838). However, their model capacities are much greater than that of TDCN. For example, EDTER, which is based on a transformer, requires two independent models with at least 300M parameters. Its number of parameters is hundreds of times that of the proposed model, which may significantly hinder its practical application. Considering that the boundary detection task is more of a pretask and serves subsequent possible visual tasks, ignoring the model capacity is inappropriate. These details are analyzed in the following section.

Next, the method was evaluated using the NYUD. Specifically, it can be divided into two subdatasets. One is RGB images, and the other is based on HHA images that indicate the scene depth. TDCN was evaluated on both sets of images. Some comparison methods were also considered. The results are shown in Table 3.

**Table 3:** Performances on NYUD.

Method	ODS	OIS	ODS	OIS	ODS	OIS
gPb-UCM [38]	.632	.661				
gPb-NG [69]	.687	.716				
SE [63]	.695	.708				
SE+NG+ [70]	.710	.723				
	RGB		HHA		RGB-HHA	
HED [9]	.720	.734	.682	.695	.746	.761
CHRNet [44]	.729	.745	.718	.731	.750	.774
PiDiNet [33]	.733	.747	.715	.728	.756	.773
LPCB [12]	.739	.754	.707	.719	.762	.778
RCF [43]	.743	.757	.703	.717	.765	.780
AMH-Net [68]	.744	.758	.716	.729	.771	.786
BDCN [13]	.748	.763	.707	.719	.765	.781
DiffusionEdge [32]	.761	.766				
EDTER [31]	.774	.789	.703	.718	.780	.797
TDCN	.737	.752	.716	.730	.758	.777

Table 3 lists the performances on RGB and HHA images. The RGB-HHA column gives the results based on the averaged prediction from RGB and HHA images. First, for the RGB images, TDCN achieved scores of 0.737 and 0.752 in ODS and OIS, respectively. For the HHA images, the corresponding values were 0.716 and 0.730. The performance from RGB to HHA images decreased. In fact, not only the proposed method but all methods have similar situations. This is because it is more difficult to determine the boundary from HHA images. However, compared with the other methods, the degradation for TDCN is less, about −0.021. This is mainly because of its good performance on the HHA images. In general, TDCN is competitive for indoor boundary detection as well as for the natural landscape images discussed above.

TDCN is initialized from draft. In contrast to most other methods, the backbone is derived from mature, well-known structures. For example, the initialization of RCF is based on VGG16, trained on ImageNet. When training on a boundary detection dataset, the parameters must be fine-tuned at a small learning rate. For TDCN, because there are no pioneering studies, the parameters are randomly initialized. This is valuable because pretraining on other large datasets incurs additional costs. Moreover, one can avoid problems caused by dataset incompatibility. This may explain why EDTER, which is initialized by a pretrained ViT and has outperformed others on RGB images, does not rank well on HHA images. In contrast, TDCN treats different datasets equally without preference. Auxiliary datasets and pretraining are unnecessary. It can adapt to new problems more quickly and effectively, even if there have never been relevant problems before. This characteristic may be valuable in solving emerging problems.

### 4.4 Lightweight and universal boundary prediction

**Fig 9:**
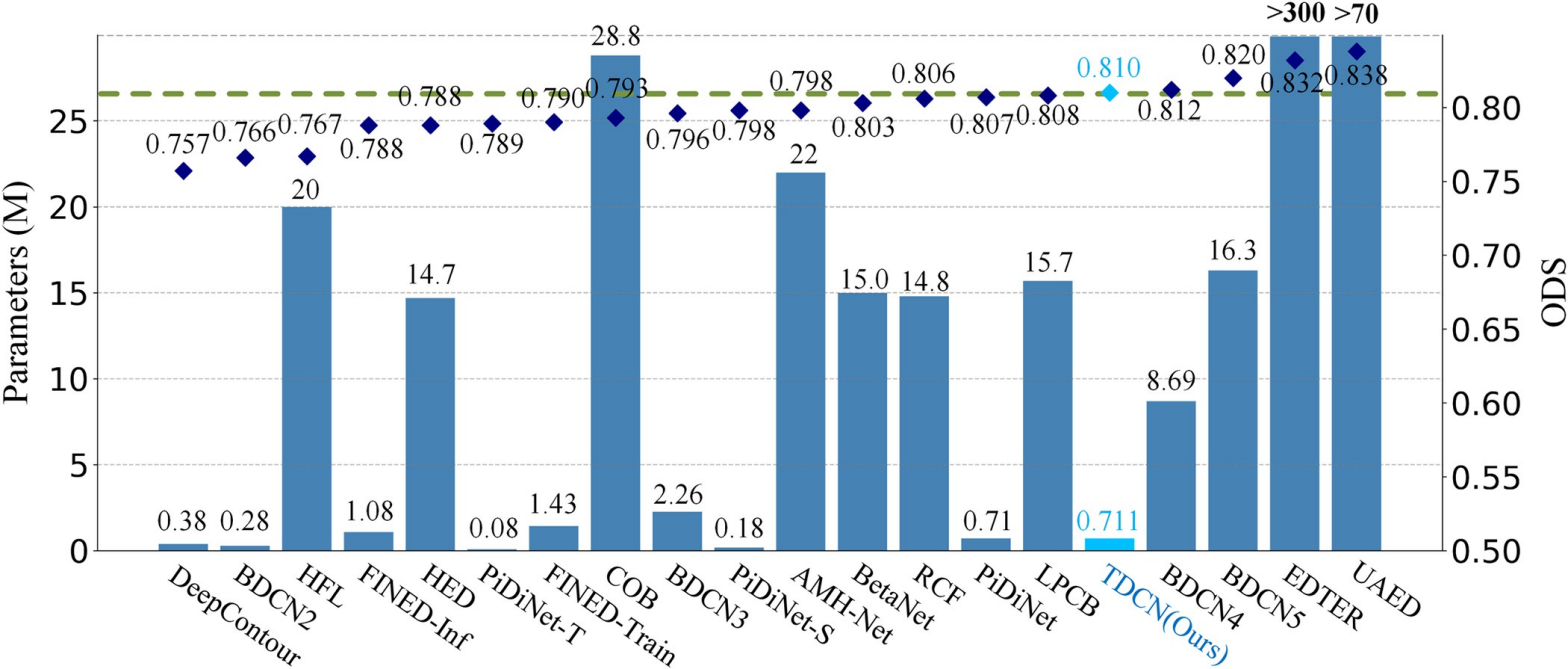
Number of parameters and corresponding performances (ODS) of different structures.

TDCN is lightweight and unified. First, it is lightweight and has lower memory and computational power consumption. To illustrate this point better, the number of parameters of the models and their performances are shown in Fig 9.

Fig 9 shows the performances of the methods and the number of required training parameters. A basic trend is that, the more the parameters, the better the performance. For example, for the same structure (BDCN), BDCN5 with five ID blocks (incremental detection blocks, proposed in BDCN) performed much better than BDCN2 with only two ID blocks. Predictably, the number of training parameters required by BDCN5 increased from 0.28M to 16.3M. This trend also occurred for PiDiNet, where different suffixes (S: half of the channel numbers, T: one-third of the channel numbers) represent the number of channels. This is because, in general, model capacity is related to learning ability. Additional parameters indicate a stronger capability for learning mapping.

However, the proposed method achieves better performance with fewer parameters. The parameters to be learned in the model are only 711k, which is only one-twentieth of that of BDCN5 and 1% those of EDTER and UAED, with the best performance (ODS-838). Unlike most methods that stack a large number of normal convolutional layers (BDCN and UAED) or vanilla self-attention layers (EDTER and DiffusionEdge), the proposed lightweight model achieves effective boundary detection and competitive performance. This can be attributed to the specific design. Inspired by the pixel difference convolution [33] and dilation configuration, the TAG layers in the dilation mode can capture richer absolute gradient information based on the Sobel, Laplace, and angular operators. This provides a strong inductive bias for the boundary detection task and effective token embedding for the subsequent transformer. By explicitly introducing a gradient operator, this design improves the efficiency of feature extraction. The transformer with boundary-aware attention also helps obtain valuable boundary information. Facilitated by this, there are only four transformer blocks in TDCN, whereas other methods often require more blocks. In summary, fusing richer prior knowledge (gradient and boundary information) and technical design (depthwise separable convolutions) ensures the effectiveness and light weight of the model.

**Fig 10:**
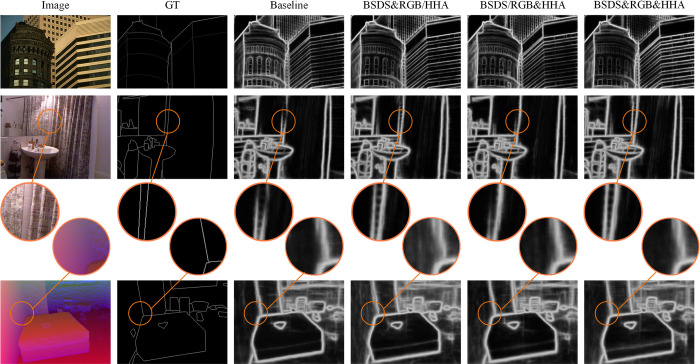
Prediction results for BSDS (top), NYUD-RGB (middle), and NYUD-HHA (bottom).

Moreover, the performance of TDCN in unified boundary prediction was investigated. In this study, a unified model was used to train multiple datasets simultaneously and provide the prediction results. As mentioned earlier, three datasets were used: BSDS, NYUD-RGB, and NYUD-HHA. The first two are based on RGB images, and the last is based on HHA images. Experiments were conducted using multiple combinations. The quantitative indicators and prediction results are shown in Table 4, and Fig 10.

**Table 4:** Performance comparison between unified prediction and dataset-specific prediction.

Dataset	BSDS		RGB		HHA	
	ODS	OIS	ODS	OIS	ODS	OIS
Baseline	.810	.826	.737	.752	.716	.730
BSDS&RGB	.808	.825	.742	.757	-	-
BSDS&HHA	.811	.826	-	-	.708	.724
RGB&HHA	-	-	.736	.751	.716	.731
BSDS&RGB&HHA	.806	.824	.740	.756	.708	.722

In Fig 10, three images selected from BSDS, NYUD-RGB, and NYUD-HHA are shown. The title “baseline” means the dataset-specific version TDCN. “BSDS&RGB/HHA” refers to the first two images predicted from BSDS&RGB TDCN, and the third image is predicted from BSDS&HHA TDCN. Similarly, “BSDS/RGB&HHA” refers to the first image predicted from BSDS&HHA TDCN, and the last two images are predicted from RGB&HHA TDCN. The final column combines all three datasets.

Fig 10 and Table 4 reveal that the unified prediction maintains a high performance. For example, the unified prediction trained on the BSDS&RGB dataset for BSDS achieved scores of 0.808 in ODS and 0.825 in OIS, which were only 0.002 worse than the baseline. The ODS for NYUD-RGB clearly improved from 0.737 to 0.742 compared with the baseline. For the unified model trained on the RGB&HHA dataset, the corresponding performance was also competitive. A higher score of 0.811 in ODS was achieved for the BSDS dataset when using BSDS&HHA TDCN. However, the scores for the HHA dataset under BSDS&HHA and BSDS&RGB&HHA TDCN decreased significantly. They degenerated to 0.708, which is approximately 0.008 below the benchmark (0.716). This may result from the distribution shift caused by the large gap between the involved BSDS and HHA datasets compared with BSDS&RGB sharing the same channels (RGB) and RGB&HHA focusing on the same scene (indoors), as shown in Fig 10.

Overall, the proposed method was effective in predicting multiple datasets simultaneously using a unified model. This can be attributed to the dataset tokens. They made it possible to train and predict multiple datasets simultaneously. When inputting images from different domains, the corresponding dataset token embedding was used to calculate the similarity in boundary-aware attention. The change in the number of parameters brought about by such a design was very small (approximately 1k). This is negligible compared with the retraining of a new model. This indicates that the features extracted using the proposed model are general and effective. If the datasets are closely related, the model performance can even be improved. Because boundary detection is a fundamental vision task, obtaining a general model based on deep learning is meaningful. This demonstrates the good characteristics of TDCN.

### 4.5 Ablation study and parameter sensitivity

**Fig 11:**
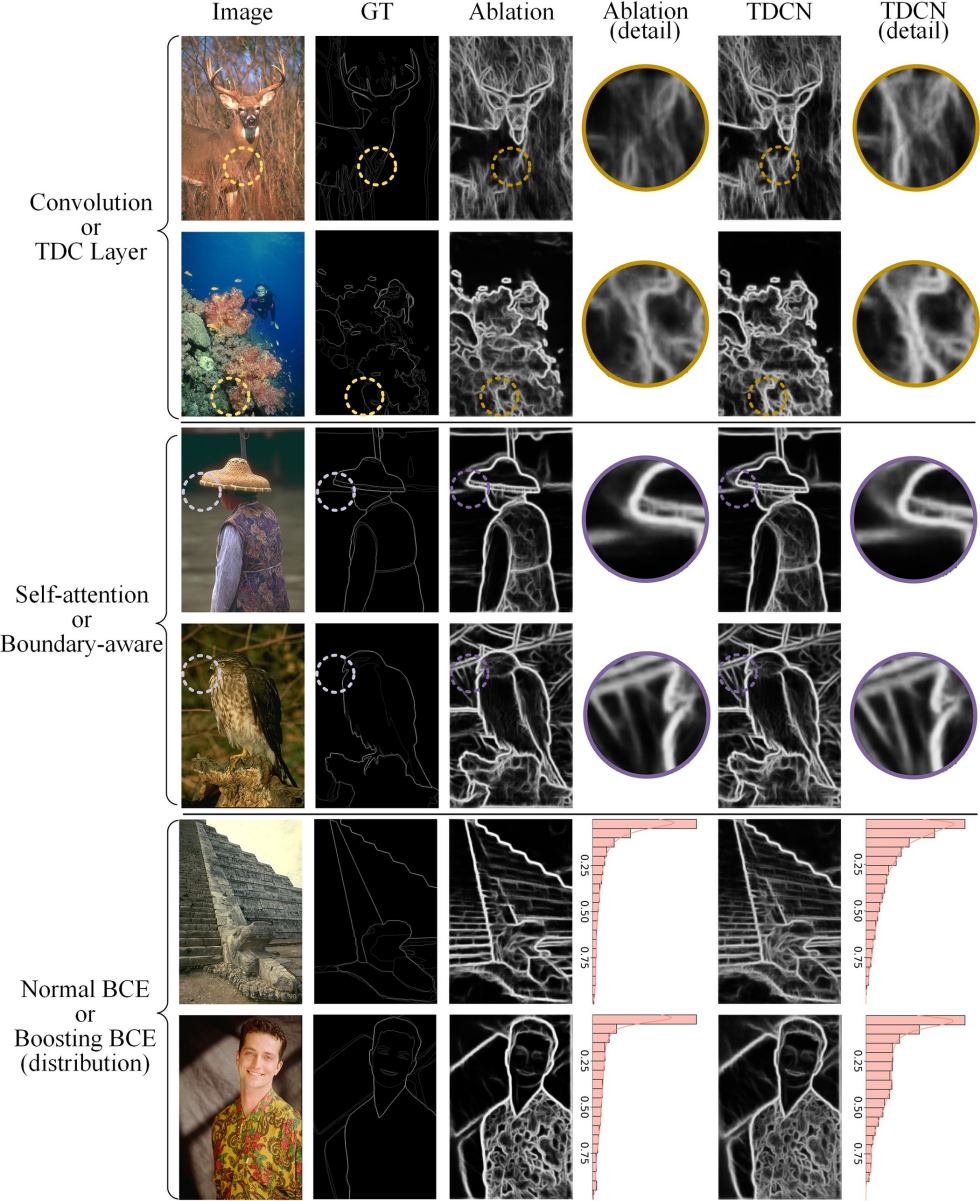
Prediction results under complete TDCN and corresponding ablation study after three epochs.

In this section, the effect of each component and the sensitivity of the hyperparameters are analyzed. TDCN consists of a difference convolution, a transformer, and head functions. To adapt to the boundary detection task, these three parts involve the TAG layer, boundary-aware attention, and boosting training strategies, respectively. Subsequently, their specific effects and values are analyzed based on an ablation study. The performances of the different combinations and prediction results are presented in Table 5 and Fig 11, respectively.

**Table 5:** Ablation study on TAG layer, boundary-aware attention, and boosting strategy after three training epochs.

W/o TAG	W/o BAtte	W/o Boost	ODS	OIS
×	×	×	.787	.809
×	×	√	.788	.812
×	√	×	.795	.816
√	×	×	.799	.818
×	√	√	.797	.815
√	×	√	.799	.819
√	√	×	.800	.820
√	√	√	.805	.821

Multiple cases are presented in Table 5 and Fig 11. The scores were evaluated using the BSDS dataset after three training epochs. In Table 5, the checkmarks indicate whether the relevant technology was adopted. For a fair comparison, cross marking does not simply mean discarding the corresponding technology. It is replaced with a competitive competitor. Specifically, the counterpart of the TAG layer is the normal convolution layer, the counterpart of boundary-aware attention is the typical self-attention mechanism, and the counterpart of the boosting strategy is the traditional cross entropy in the parallel mode. Similarly, in Fig 11, the column with title “Ablation” is for the ablation study. The first two rows correspond to the fifth row of Table 5, the middle two correspond to the sixth row of Table 5, and the last two correspond to the seventh row of Table 5.

For the designed difference convolution, the upper part of Fig 11 shows the prediction results and corresponding details using traditional convolution and the TAG layer, respectively. Intuitively, compared with traditional convolution, the boundary predictions of the TAG layer are more obvious and significant. Particularly in the detailed images, the TAG layer provides a higher response for the boundaries around the noisy background. This improvement can be attributed to the difference in the convolution integrated in the TAG layer. Unlike traditional convolution, differential convolution uses the gradient of the feature map as the input instead of directly feeding the original. Although vanilla convolution layers can gradually capture the gradient information through a hierarchical structure, explicit learning based on gradient information is undoubtedly faster. When pursuing a lightweight model, the TAG layer can directly learn the mapping from the gradient to the boundary without stacking additional layers, which is clearly a more economical method.

Second, for the proposed attention layer, the middle part of Fig 11 shows the prediction results and the corresponding details using traditional self-attention and the proposed boundary-aware attention, respectively. Here, two pictures with blurred backgrounds were deliberately chosen to demonstrate the differences between the two attention mechanisms. In the detailed picture, even the out-of-focus boundaries can be predicted effectively by the proposed attention layer. More importantly, compared with traditional attention, the predicted boundaries are more continuous. This phenomenon was related to the similarity measurement method of the proposed attention layer. For boundary-aware attention, the output is a weighted sum of patch-boundary pairs rather than based on patch-patch pairs. Its essence is to strengthen the correlation between the patch and the elongated area using several preset directional filters. Considering that the boundaries are always continuous, such adjustments are more conducive to improving the consistency of the boundaries.

Finally, for the boosting strategy in the proposed head function, the prediction results were compared with the traditional cross-entropy methods for the ablation study in the lower part of Fig 11. To demonstrate the characteristics of the boosting strategy better, a histogram of the pixel-level prediction probabilities is also displayed on the right side of each prediction result. Overall, the normal BCE method based on the Adam optimizer produced overconfident results. In contrast, the prediction results of boosting BCE appear more moderate, which may lead to reasonable confidence. This can be attributed to the specific design of the head functions. Unlike conventional methods that treat head functions as multiple independent learners, the proposed model was constructed in the form of ensemble learning based on multiscale learners. Using the designed bidirectional boosting strategy, learners of different scales can fully utilize their respective abilities to improve the boundary detection effects.

From a quantitative view from Table 5, there is a huge gap in performances when determining whether to adopt the three technologies. When it is a vanilla CNN-transformer network based on normal convolution, self-attention, and cross entropy, it only achieves scores of 0.787 in ODS and 0.809 in OIS. In this mode, no special design provides valuable prior knowledge related to the boundary detection tasks. However, with the introduction of the three proposed technologies, this capability becomes stronger. In particular, with the addition of the TAG layer and boundary-aware attention, the ODS scores increase to 0.799 and 0.795, respectively. The performances can be further improved by combining these technologies. When all three techniques are employed, the model achieves scores of 0.805 for ODS and 0.821 for OIS, requiring only three epochs.

Finally, the hyperparameters were analyzed. Here, *ρ* in Eq (10) and *α* in Eq (15) were mainly considered. These parameters are related to the loss function. The former controls interactions between different head functions. The larger *ρ* means that the subsequent head function assigns greater weights to those misclassified samples. The coefficient *α* is used to balance the boosting loss $L_{Boost}^{s}$ and attention loss $L_{Atten}^{s}$. The parameter sensitivity was analyzed separately, as shown in Table 6.

**Table 6:** Parameter sensitivity analysis for TDCN after three training epochs.

*ρ*	*α*	ODS	OIS
0.2	20	.802	.821
0.6	20	.803	.822
0.8	20	.803	.821
1.0	20	.804	.822
0.4	0	.801	.819
0.4	10	.804	.822
0.4	50	.804	.824
0.4	100	.803	.823
0.4	200	.801	.821
0.4	20	.805	.821

As shown in Table 6, the quantitative indices listed in Table 6 were evaluated using the BSDS dataset after three training epochs. Table 6 shows that the effect of parameter *ρ* on the performance was not significant. Although the optimal configuration (*ρ* = 0.4) obtained a score of 0.805 in ODS and a score of 0.821 in OIS, the scores of other configurations were also relatively high, such as 0.804 when setting *ρ* = 1.0. This shows that the proposed model is not sensitive to parameter *ρ*. Under the boosting strategy, the method can achieve good performance for a large range of *ρ*.

In contrast, the influence of parameter *α* is fairly obvious. A rough trend is that, with the increase in *α*, the ODS increases from 0.801 (*α* = 0) to 0.805 (*α* = 20) and then decreases to 0.801 (*α* = 200). In essence, *α* is used to balance the two loss functions adopted. This determines how to guide model learning and the corresponding preferences. Therefore, this parameter is closely related to performance. When setting *α* = 20, as discussed previously, the scores are the highest. Choosing too large or too small degrades performance. This also reveals that these two losses are important and cannot be replaced by each other. The $L_{Atten}^{s}$ derived from the proposed boundary-aware attention improves the effectiveness of the feature extraction stage. The $L_{Boost}^{s}$ involved in the head functions enable complicated boundaries to be detected correctly, whether for large- or small-scale boundaries. They work together to ensure the good performance of the method.

### 4.6 Limitations and future work

In this study, a novel transformer structure was designed with difference convolution for lightweight and universal boundary detection. In the experiments, although the method achieved significant advantages among methods with similar model capacities, there was still a performance gap with larger models. In addition to the obvious reasons, it is believed that this may be related to parameter initialization. The backbone was specifically designed for the boundary detection task, and the parameters of the model did not undergo pre-training on large datasets. Perhaps introducing self-supervised learning would be a good way to improve the performance. Furthermore, to the best of the authors’ knowledge, the loss in a conventional transformer generally appears only in the output layer of the model. The attention mechanism is mostly used to observe whether the trained model captures an effective visual representation without human intervention. Instead, in this study, a novel loss function was designed to guide model training directly, which is one of the contributions of the proposed method. This loss might be adapted to other visual tasks (such as semantic segmentation) in addition to boundary detection tasks after adjustment. Further research is needed to extend the proposed attention loss to more potential scenarios and explore ways to improve model performance. Finally, the proposed method is lightweight and unified, meeting the realistic requirements of low-level vision tasks. Owing to a lack of engineering development experience, the current model is still in the theoretical research stage. Considering that the method requires less memory and computing power, it is significant to deploy the model on edge devices, which will be the focus of planned future work.

## 5. Conclusion

An effective boundary detection network, TDCN, based on a transformer was proposed. Unlike a pure transformer, it involves a difference convolution when acquiring the token embedding. Difference convolution, including the TAG layer, explicitly extracts the gradient information closely related to boundary detection. These features were further transformed together with the dataset token through the proposed transformer. The boundary-aware attention in the transformer and the TAG layer in the convolution achieve efficient feature extraction to keep the model lightweight. Moreover, dataset token embedding gives the model universal prediction capability for multiple datasets. Finally, a bidirectional boosting strategy was used to train the head functions for the multiscale features. These strategies and designs ensure good model performance. Multiple experiments demonstrated the effectiveness of the method. This study represents a novel attempt at solving the fundamental vision task of boundary detection based on transformers.
